# Use of Complementary and Alternative Medicine among People with Multiple Sclerosis in the Nordic Countries

**DOI:** 10.1155/2012/841085

**Published:** 2012-12-11

**Authors:** L. Skovgaard, P. H. Nicolajsen, E. Pedersen, M. Kant, S. Fredrikson, M. Verhoef, D. W. Meyrowitsch

**Affiliations:** ^1^Department of Public Health, University of Copenhagen, 1014 Copenhagen, Denmark; ^2^The Danish MS Society, 2500 Valby, Denmark; ^3^NAFKAM, University of Tromsø, 9037 Tromsø, Norway; ^4^Department of Neurology, Akershus University Hospital, 1474 Nordbyhagen, Norway; ^5^Department of Neurology, University of Southern Jutland, 7100 Vejle, Denmark; ^6^Department of Clinical Neuroscience, Karolinska Institute, 14186 Stockholm, Sweden

## Abstract

*Aims*. The aim of the study was to describe and compare (1) the types and prevalence of complementary and alternative medicine (CAM) treatments used among individuals with multiple sclerosis (MS) in the Nordic countries; (2) the types of conventional treatments besides disease-modifying medicine for MS that were used in combination with CAM treatments; (3) the types of symptoms/health issues addressed by use of CAM treatments. *Methods.* An internet-based questionnaire was used to collect data from 6455 members of the five Nordic MS societies. The response rates varied from 50.9% in Norway to 61.5% in Iceland. *Results*. A large range of CAM treatments were reported to be in use in all five Nordic countries. Supplements of vitamins and minerals, supplements of oils, special diet, acupuncture, and herbal medicine were among the CAM treatment modalities most commonly used. The prevalence of the overall use of CAM treatments within the last twelve months varied from 46.0% in Sweden to 58.9% in Iceland. CAM treatments were most often used in combination with conventional treatments. The conventional treatments that were most often combined with CAM treatment were prescription medication, physical therapy, and over-the-counter (OTC) medications. The proportion of CAM users who reported exclusive use of CAM (defined as use of no conventional treatments besides disease-modifying medicine for MS) varied from 9.5% in Finland to 18.4% in Norway. In all five Nordic countries, CAM treatments were most commonly used for nonspecific/preventative purposes such as strengthening the body in general, improving the body's muscle strength, and improving well-being. CAM treatments were less often used for the purpose of improving specific symptoms such as body pain, problems with balance, and fatigue/lack of energy. *Conclusions*. A large range of CAM treatments were used by individuals with MS in all Nordic countries. The most commonly reported rationale for CAM treatment use focused on improving the general state of health. The overall pattern of CAM treatment use was similar across the five countries.

## 1. Introduction

Multiple sclerosis (MS) is a severe neurological disease, characterized by chronic course of exacerbation and remission of symptoms, leading to severe disability. The absolute number of individuals with MS is increasing in the western countries and represents a substantial challenge to treatment, prevention, health promotion, and rehabilitation. The causes of MS are still unknown [1, 2].

Individuals with MS face many challenges in their everyday life, like many other groups of people with chronic illness. There is no cure for MS, the medical treatment options are limited for some types of MS, and treatments often have many side effects. In addition, MS is often characterized by a wide range of accompanying symptoms [1, 3].

People with MS are widely using complementary and alternative medicine (CAM) treatments [4–21]. Internationally, study results indicate that the prevalence of CAM use among people with MS ranges from 41% in Spain to 70% in Canada and 82% in Australia [8, 21, 22]. The reasons for CAM use vary from treatment of concrete symptoms [4, 5, 13, 23, 24] to bodily exploration and development of coping strategies [18, 25–28], and CAM treatments are most often used in combination with conventional treatment [17, 22, 29, 30]. In Denmark, results of previous small-scale and often unpublished studies suggest that the prevalence of CAM treatment use is fairly consistent among individuals with MS, ranging from 48% (1998) and 54% (2002) to 52% (2007) [4–6] (The studies are not entirely comparable due to differences in the wording of the survey questions.). In these studies, treatment with CAM was primarily used to relieve pain, fatigue, and problems with balance and walking [4, 5]. A Norwegian study showed that CAM was also used for nonspecific purposes by people with MS and as part of overall self-care management [25]. However, little is known about the general use of CAM among people with MS in the Nordic countries; there are limited data on types and prevalence of CAM treatments used, on types of conventional treatments that CAM treatments are used in combination with, and on the overall symptoms/health issues which form the individual rationale for use of CAM treatments. Therefore, the aim of this study was to collect such information, and in 2010, the five Nordic MS societies initiated a research project, investigating the use of CAM among their members. In this paper, we present the results of a survey which was conducted simultaneously in all five Nordic countries.

## 2. Material and Methods

The survey used an internet-based questionnaire and was conducted during the period from April 2011 to June 2011. Based on knowledge from a large Danish research project that took place from 2004 to 2010 within the Danish Multiple Sclerosis Society and investigated treatment collaboration between conventional and complementary practitioners [27, 28, 31–33], a questionnaire was developed, tested, and validated using cognitive interviews as well as assessments by an expert group of Nordic neurologists [34]. The questionnaire was developed in Danish and translated into the Nordic languages, then back to Danish by health professionals with Danish or the other Nordic languages as native language/second language, respectively. A synthesis of the original and the retranslated Danish versions was produced for each Nordic language, and the questionnaires were adjusted. The questionnaire was programmed in IBM Inquisite ASP, using skip sections and branching and thereby ensuring ease of response for a variety of respondents.

As previous Nordic studies had shown a lack of consensus regarding the definition of CAM [35–37], a questionnaire was developed to collect information about both the use of conventional as well as CAM treatments. The terms conventional or CAM treatment were not used in the questionnaire, to avoid response bias related to these labels. This strategy also provided the possibility of investigating the use of conventional and CAM treatments in combination. For each treatment, participants were asked whether they used it and what their motives for use were. Use of disease-modifying medicine for MS was not included in the study.

Based on power calculations and expected dropout due to members who were deceased, lived abroad, or did not have MS (registration error), 1050 people with MS were selected randomly from the member registers of the Swedish, Norwegian, and Finnish MS societies, and 3500 people with MS were selected randomly from the member register of the Danish MS society. In Iceland, the sample included the total number of all individuals who appeared in the member register of the national MS society (In Iceland, it has not been possible to distinguish between members with MS and supporting members in the register. Letters were therefore sent to all members of the Icelandic MS society (*n* = 780), asking only people with MS to answer the questionnaire.).

A letter with a personal code was sent to all respondents, asking them to fill out the questionnaire online. Receiving the questionnaire in paper form was not an option. Reminders to nonrespondents were sent twice. As shown in Table 1, the response rates varied from 50.9 to 61.5.

In Iceland, it was not possible to distinguish between MS Society members with MS and supporting members. Therefore, an analysis of representativeness could not be carried out. Keeping this in mind, the results of the Icelandic data are still presented in the subsequent sections.

Comparative analyses included use of specific CAM modalities as well as specific symptoms/health issues addressed by CAM users as rationale for use. As none of the five countries constitute a natural a priory reference, we have found it most correct to employ a changing reference. The country with the lowest prevalence of a specific variable was hence used as reference for presenting odds ratios (ORs), indicating the comparative relations for each variable. *P* values have not been included in the tables due to risk of visual complexity, but statistical significance has been determined by interpretation of 95% confidence intervals (CIs) and is marked by “+” in the tables. As the choice of performing multiple comparisons entails the risk of mass significance and thereby finding statistical significance that are due to random error rather than real differences, we have been highly aware of interpreting significant differences in single variables in an overall perspective.

The definition of CAM treatments was based on the National Center for Complementary and Alternative Medicine's (NCCAM) definition of CAM as “a group of diverse medical and health care systems, practices, and products that are not generally considered part of conventional medicine” [38]. The specific CAM treatment modalities used in the questionnaire were chosen on the basis of known prevalence of use in the Nordic countries, and room was left open for addition of nonpredefined modalities by respondents.

## 3. Results

### 3.1. Representativeness

Analyses of representativeness showed no major differences regarding distribution of gender and age (it was not possible to procure data on other variables than gender and age) between the national member registers and the samples. However, as shown in Table 1, the participants of <40 years and those of >60 years were slightly underrepresented when comparing respondents with sample groups. Participants of 41–60 years were consequently slightly overrepresented. These differences were borderline significant.

### 3.2. CAM Treatment Modalities in Use

The total prevalence of CAM use within the last 12 months varied from 46.0% in Sweden to 58.9% in Iceland. The difference was borderline significant (*P* = 0.059). The prevalence was 51.8% in Denmark, 52.7% in Norway, and 55.6% in Finland.

Table 2 presents the types and prevalence of CAM treatments used by individuals with MS in the Nordic countries. Up to 29 different CAM treatment modalities were reported to be used. The prevalence of the most commonly reported treatments was quite similar among the five Nordic countries.

In all five countries, supplements of vitamins and minerals, supplements of oils, special diet, acupuncture, herbal medicine, reflexology (reflexology, also called zone therapy, involves the physical act of applying pressure to the feet. It is based on what reflexologists claim to be a system of zones and reflex areas that they say reflect an image of the body on the feet with the premise that such work effects a physical change to the body), yoga, and meditation were among the most commonly used CAM modalities, though with some variation in the order of importance. Alternative types of massage (shiatsu massage and healing massage), craniosacral therapy, healing, homeopathy, amalgam removal, qi gong, and naprapathy were used by more than 5% in one or more countries.

When comparing the use of specific CAM modalities in the five Nordic countries, some significant differences were seen (see Table 2). The use of CAM in the two countries with the highest prevalence of total use, Finland and Iceland, differed with regards to the types of CAM modalities in use. Finland was characterized by a high-level use of supplements and a low use of energetic CAM modalities (by energetic CAM modalities, we mean modalities that work with energies in the human body in ways that transcend the principles of conventional science of nature) such as healing and craniosacral therapy, whereas Iceland was characterized by a high-level use of energetic CAM modalities such as reflexology, craniosacral therapy, and healing and a low use of supplements. Acupuncture was a popular CAM modality in all five countries, though with a lower use in Finland. The use of reflexology was widespread in Denmark, and the use of qi gong was widespread in Sweden when compared with the other Nordic countries.

### 3.3. Combination of Conventional Treatment and CAM Treatment

The types and prevalence of conventional treatment modalities, besides disease-modifying medicine for MS, which were reported to be used in combination with CAM treatment are presented in Table 3 (The use of disease-modifying medicine for MS is not included in the study and hence not in the table.).

Within the last 12 months, CAM treatments were most commonly used in combination with conventional treatment in all five Nordic countries. The prevalence of exclusive CAM use (defined as no conventional treatments used besides disease-modifying medicine for MS) among CAM users varied from 9.5% in Finland to 18.4% in Norway. It was significantly higher in Norway and Sweden compared to lowest prevalence in Finland. Exclusive use of CAM was not directly connected to a high prevalence of total CAM use which was highest in Iceland and Finland. CAM treatment was most commonly combined with use of prescription medicine, physical therapy, and OTC medications.

The prevalence of the different conventional treatments use in combination with CAM was quite consistent among the five Nordic countries, though with some significant differences, for example, regarding the use of prescription medicine in combination with CAM treatment which was high in Finland and low in Norway. Combination of physical therapy and CAM was lowest in Sweden, and combination of OTC medications as well as therapeutic horseback riding and CAM was highest in Denmark.

### 3.4. Symptoms/Health Issues Addressed by Use of CAM Treatment

An overview of the types and prevalence of symptoms/health issues addressed by use of CAM treatment in the five Nordic countries is provided in Table 4. In all five countries, CAM treatments were primarily used for nonspecific/preventive purposes and less often with the purpose of treating specific symptoms. In all five countries, strengthening the body in general, improving well-being, preventing symptoms, and improving the body's muscle strength were reported as common reasons. Treatment of specific symptoms/health issues such as fatigue/lack of energy, body pain, problems with balance, and spasms/tensions/cramps were also commonly mentioned as reasons for CAM use. There were no significant differences between countries in relation to the total prevalence of nonspecific/preventative purposes. However, some differences existed between the countries at a single variable level, for example, in connection to the purpose of reducing the frequency of attacks which had very high prevalence in Denmark and Iceland, compared to Finland. Also, improving the body's muscle strength was common in Iceland when comparing with the other Nordic countries.

## 4. Discussion

### 4.1. Comparison with Other Studies

The present study shows a widespread use of CAM treatments among people with MS. These findings support the findings of similar studies [7, 8, 16–22, 39], where a high total prevalence as well as a large variation of CAM modalities was found. The total prevalence of CAM use among people with MS in Denmark is similar to what has been indicated by earlier minor/unpublished studies [4–6]. The prevalence of some specific CAM modalities (e.g., reflexology and craniosacral therapy) was found to be lower in this study compared to the before-mentioned Danish studies that were based on telephone interviews. The studies are not directly comparable due to differences in the time periods included, but the variation may still reflect the classic challenge of prevalence of CAM use reported to be lower in questionnaire studies compared to studies based on telephone interviews [40].

Regarding the types of CAM treatment used, this study supports previous findings where supplements of vitamins and minerals, supplements of oils, herbal medicine, special diets, acupuncture, and yoga were reported as popular CAM modalities among people with MS [8, 13, 16–18, 20, 23, 41]. However, this study contributes an important focus on the distinction between CAM/non-CAM elements within specific modalities that are usually regarded as CAM (massage) or usually regarded as conventional treatment (psychological intervention/psychotherapy). One treatment modality may occur in different versions, and these versions may vary quite a lot with regard to the medical and pathological assumptions on which they work. For example, the type of massage that is generally included in physical therapy lies within the frame of conventional medicine and is not to be regarded as a CAM treatment if following NCCAM's definition. Healing massage, on the other hand, is to be regarded as a CAM treatment as it is seldom practiced within the conventional health care system and as it transcends the principles of conventional science of nature. Such distinctions may be important to include in studies of CAM use in order to obtain accurate interpretations. One consequence of the inclusion of such distinctions in this study is that the prevalence of massage is notably lower compared to other studies as only alternative types of massage are included as CAM.

This study shows that CAM treatments are most often used in combination with conventional treatments and supports hereby the findings of similar studies [9, 17, 22, 29, 30]. The prevalence of exclusive CAM use is comparable to what has been found in other studies where prevalence has ranged from 9.4% [9] to 29% [17].

The motives for using CAM treatment among people with MS in the Nordic countries found in this study include both those of a specific and of a general nature, and the study results hereby support previous findings [8, 10, 17, 18, 20, 25, 42, 43]. However, the results of this study emphasize that general health issues constitute the most important type of motive for CAM use.

Regarding differences in CAM use in the Nordic national populations, a study from 2005, comparing use of CAM in Denmark, Norway and Sweden, showed that the prevalence of CAM use varied from 34% in Norway to 45% in Denmark and 49% in Stockholm (Sweden) [44]. The study also showed differences regarding therapy preferences. These findings are to a certain extent supported by the present study with regards to differences regarding therapy preferences. However, the present study does not support the findings of large differences in the prevalence of use of CAM treatments as shown by Salomonsen et al. (2011) and by recent national studies of CAM usage, for example, between Denmark and Norway [35]. This may be due to differences in overall study design, methods of data collection, and/or different definitions of CAM. Such challenges in comparing CAM use across national borders within EU have been stated recently by the CAMbrella project [45]. However, a Danish-Norwegian research project from 2009 showed major differences in reported use of CAM (both in terms of overall prevalence and in terms of the variety of CAM modalities in use) in Danish and Norwegian hospitals, using the same questionnaire simultaneously [35]. This may point to the fact that some differences in attitudes towards CAM exist on political/organizational levels between the Nordic countries, but that these differences cannot necessarily be generalized to specific groups of patients, for example, chronic patient groups such as people with MS.

### 4.2. Differences between the Nordic Countries

According to our knowledge, this is the first cross-national study to compare the use of CAM treatments among people with MS in the Nordic countries and one of the first studies to compare the use of CAM treatments among populations in the Nordic countries.

Although characterized by extensive accordance, the study shows some differences between the MS populations in the Nordic countries. In their study on CAM use in Denmark, Norway, and Sweden from 2005, Hanssen et al. (2008) conclude that reasons for variations in the use of CAM therapies in such culturally uniform areas, where there is also equally little financial support for CAM treatments, remain unknown [44]. Regarding the overall differences found in this study, we may state the same conclusion. There are no obvious explanations to the differences in the prevalence of total CAM use, in the prevalence of total use of energetic CAM modalities, in the prevalence of exclusive CAM use, or in the prevalence and distribution of treatment purposes addressed. We may speculate that differences within modern health politics or differences in, for example, economical, historical, philosophical, and geographical aspects, having influenced the development of the Nordic health care systems, could be important factors. Regarding differences in use of specific CAM modalities, explanatory factors exist in some cases. The high prevalence of use of therapeutic horseback riding in combination with CAM in Denmark can be explained by the integration of this modality in the rehabilitation programme at one of the Danish MS hospitals. Differences in the prevalence of use of physical therapy in combination with CAM seem to reflect the differences in access to cost-free treatment of this modality [46]. In Denmark, for instance, physical therapy is provided cost-free for all people with MS as a maintenance ongoing program, which is reflected by a high prevalence when compared with Sweden, where the financial support for physical therapy is not as generous. The prevalence of use of reflexology was high in Denmark in this study, as it has been in Danish population studies for decades [47]. This might be influenced by the development and highly profiled research of neuromuscular reflexology during the 1980s and 1990s in Denmark [48]. We have found no explanation of the high prevalence of use of qi gong in Sweden.

### 4.3. Strengths and Limitations

The study is based on a large sample of respondents, allowing for statistical tests to be performed on various levels. A further strength of the study is that surveys have been performed simultaneously in all five Nordic countries, based on a very thorough process of translation, using the same methods. The possibility of valid comparison among the countries has thereby been strengthened. The development of the questionnaire, including a pilot test of 400 Danish respondents, three sessions of cognitive interviews, and the coding of numerous skip-sections as well as extensive use of branching, has made it possible to collect data of rather high complexity among respondents with physical as well as cognitive limitations. Although we cannot be sure that all the respondents have understood the questions in the exact same way, the above-mentioned preparations have contributed considerably to the quality of the data.

At the same time, the internet-based questionnaire entails a limitation as it requires internet access. One may also suspect the internet-based questionnaire to discriminate in favor of younger respondents. As shown in Table 1, this has not turned out to be a general challenge, but the limited data on nonrespondents (constituting 38.5%–49.1%) implies a limitation regarding representative analyses. For example, data on levels of disability would have been relevant. An important limitation of the study is the lack of information about the members of the Icelandic MS Society, making it impossible to perform representative analyses. When asking about use of treatments within the past 12 months, recall bias must also be taken into consideration, not least within a group of respondents where the prevalence of cognitive challenges is higher than among the general population. The risk of selection bias in favour of CAM-use has been reduced by the fact that the survey was not presented to respondents as a CAM survey.

### 4.4. Implications

The present study indicates that CAM treatments are of significant importance among MS populations. The use of CAM was primarily reported for reasons related to the general state of health among the respondents, indicating that the usage is part of an overall coping strategy rather than a temporary or fortuitous attempt to alleviate a specific symptom. Therefore, the study can contribute to a better understanding of the widespread use of different CAM treatment modalities among people with MS, and among people with chronic illnesses in general. The study may also help to qualify communication between patients and representatives of conventional health care systems regarding motives, goals, and rationales linked to the CAM usage. Such communication is very relevant with regard to possible negative impact/risks connected to CAM treatments, for example, CCSVI surgery, amalgam removal, and supplements of vitamins and minerals.

In recent years, patient organizations as well as health care systems have brought into focus the possible relevance of addressing different groups of patients/members in different ways, acknowledging the lack of homogeneity in attitudes, opinions, and motives. In that respect, it would be relevant to investigate whether the tendencies indicated by this study in connection to the national groups of Nordic MS CAM users are generally applicable in each country or whether different subgroups of CAM users exist and differ from each other—and from CAM nonusers—in terms of CAM modalities used and motives for use. Further studies may elucidate these matters.

## 5. Conclusions

The results of the present study indicate that the use of CAM treatment among individuals with MS was widespread in all five Nordic countries. Interestingly, the five countries had quite similar patterns in relation to prevalence of CAM use, the types of CAM modalities used, the types of conventional treatments that CAM treatment was used in combination with, and the types of symptoms/health issues that were most often addressed by use of CAM treatment. Some differences were found between the countries as well, especially regarding the prevalence of use of some specific CAM modalities, the prevalence of exclusive CAM use, and the prevalence of use of one or more energetic CAM treatments. Generally, Iceland and Finland represented the largest differences. The analyses showed that Iceland was characterized by a high prevalence of overall CAM use, including high-level use of energetic CAM modalities, and that the CAM treatments were very often used for nonspecific/preventive purposes. Finland was also characterized by a high prevalence of total CAM use, but mainly due to a high-level use of supplements. The use of energetic CAM modalities was low in Finland compared to the other four countries, and CAM treatments were very often used in combination with prescription medicine. Patterns of use were quite homogenous between Denmark, Norway, and Sweden, with Sweden having a slightly lower prevalence of total CAM use, including the use of supplements as well as energetic CAM modalities. Norway and Sweden had higher prevalence of exclusive CAM use than the other three countries.

The results of the present study support the findings of previous, similar studies with regard to the prevalence of CAM use and the motives for CAM use among people with MS. No previous studies have compared use of CAM treatments among the five Nordic countries, and the study contributes new knowledge in this area concerning the use of CAM as well as concerning the use of CAM in combination with conventional treatments. The study is based on a large sample of respondents and may contribute to a better understanding of the role that CAM treatments play in the disease coping among people with MS as well as among people with chronic illnesses in general.

## Figures and Tables

**Table 1:** Representative characteristics of the study population.

		Denmark			Norway			Sweden			Finland			Iceland
			OR	(CI)		OR	(CI)		OR	(CI)		OR	(CI)
Member register (sex and age known)	*n* =	7123			4593			2435			5845			384*
	Male *n* = (%)	2078 (29.2)			1321 (28.8)			579 (23.8)			1514 (25.9)			Data missing
	<40 years *n* = (%)	1404 (19.7)			862 (18.8)			362 (14.9)			1224 (20.9)			Data missing
	41–60 years *n* = (%)	3607 (50.6)			2370 (51.6)			1251 (51.4)			3070 (52.6)			Data missing
	>60 years *n* = (%)	2112 (29.7)			1361 (29.6)			821 (33.7))			1551 (26.5)			Data missing

Survey sample (OR versus member register)	*n* =	3361			1014			1046			1045			384*
	Male *n* = (%)	976 (29.0)	0.99	(0.91–1.08)	291 (28.7)	0.99	(0.86–1.15)	251 (24.0)	1.00	(0.85–1.19)	274 (26.2)	1.01	(0.87–1.16)	Data missing
	<40 years *n* = (%)	671 (19.9)	1.01	(0.91–1.12)	189 (18.6)	0.99	(0.83–1.17)	157 (15.0)	1.01	(0.82–1.23)	222 (21.3)	1.01	(0.86–1.18)	Data missing
	41–60 years *n* = (%)	1690 (50.3)	0.99	(0.92–1.06)	520 (51.3)	0.99	(0.88–1.11)	536 (51.3)	0.99	(0.88–1.12)	553 (52.9)	1.01	(0.90–1.12)	Data missing
	>60 years *n* = (%)	1001 (29.8)	1.00	(0.92–1.09)	305 (30.1)	1.01	(0.88–1.16)	353 (33.7)	1.00	(0.86–1.15)	270 (25.8)	0.97	(0.84–1.12)	Data missing

Respondents (OR versus survey sample)	*n* = (response rate)	1865 (55.5)			516 (50.9)			627 (59.9)			551 (52.7)			236 (61.5)*
	Male *n* = (%)	520 (27.9)	0.96	(0.85–1.08)	150 (29.0)	1.01	(0.81–1.26)	149 (23.8)	0.99	(0.79–1.24)	138 (25.0)	0.95	(0.75–1.20)	54 (22.8)
	<40 years *n* = (%)	351 (18.8)	0.94	(0.81–1.08)	76 (14.7)	0.79	(0.59–1.05)	81 (12.9)	0.86	(0.64–1.14)	103 (18.7)	0.87	(0.68–1.13)	62 (26.2)
	41–60 years *n* = (%)	1047 (56.1)	1.11	(1.01–1.22)^+^	304 (58.9)	1.14	(0.96–1.37)	373 (59.5)	1.16	(0.98–1.36)	323 (58.6)	1.11	(0.93–1.31)	137 (57.8)
	>60 years *n* = (%)	468 (25.1)	0.84	(0.74–0.95)^−^	136 (26.4)	0.87	(0.69–1.10)	173 (27.6)	0.81	(0.66–1.01)	125 (22.7)	0.88	(0.69–1.11)	38 (16.0)

*The precise number of people with MS who are members of the Icelandic MS Society is not known. It is known that there are 430 people with MS in Iceland, and a study has shown that out of 358 people with MS, 320 (89.4%) were members of the Icelandic MS Society. Therefore, it is assumed that there are approximately 384 people with MS in the member register of the Icelandic MS Society.

^+^signifies that the prevalence is significantly higher than the lowest prevalence. Significance has been determined by interpretation of the 95% confidence intervals (CIs) (if 1 is contained in the CI, *P* < 0.05).

**Table 2:** CAM treatment modalities used by people with MS in the Nordic countries.

CAM modality	Denmark (*n* = 967)				Norway (*n* = 272)				Sweden (*n* = 288)				Finland (*n* = 306)				Iceland (*n* = 139)			
	*n*	(%)	OR	(CI)	*n*	(%)	OR	(CI)	*n*	(%)	OR	(CI)	*n*	(%)	OR	(CI)	*n*	(%)	OR	(CI)
Supplements of vitamins and minerals	759	(78.5)	1.36	(1.02–1.82)^+^	181	(66.5)	1.16	(0.83–1.61)	171	(59.3)	1.03	(0.74–1.44)	244	(79.7)	1.39	(1.00–1.91)^+^	80	(57.5)	1	
Supplements of oils	423	(43.7)	1.74	(1.18–2.56)^+^	104	(38.2)	1.52	(0.98–2.34)	88	(30.6)	1.21	(0.78–1.89)	123	(40.2)	1.60	(1.04–2.44)^+^	35	(25.2)	1	
Special diet	181	(18.7)	1.53	(0.90–2.60)	40	(14.7)	1.20	(0.66–2.20)	49	(17.0)	1.39	(0.77–2.50)	57	(18.6)	1.52	(0.86–2.71)	17	(12.2)	1	
Acupuncture	151	(15.6)	2.39	(1.47–3.88)^+^	51	(18.7)	2.87	(1.67–4.93)^+^	34	(11.8)	1.81	(1.12–3.21)^+^	20	(6.5)	1		29	(20.8)	3.19	(1.75–5.84)^+^
Herbal medicine	112	(11.6)	1.87	(1.13–3.08)^+^	31	(11.4)	1.84	(1.01–3.32)^+^	31	(10.7)	1.73	(0.96–3.14)	19	(6.2)	1		18	(12.9)	2.09	(1.06–4.10)^+^
Reflexology	107	(11.1)	7.52	(2.75–20.60)^+^	4	(1.5)	1		9	(3.1)	2.13	(0.65–6.98)	14	(4.5)	3.11	(1.01–9.57)^+^	15	(10.8)	7.34	(2.39–22.53)^+^
Yoga	106	(10.9)	1.40	(0.88–2.22)	25	(9.2)	1.17	(0.65–2.10)	42	(14.6)	1.86	(1.10–3.15)^+^	24	(7.8)	1		32	(23.0)	2.94	(1.67–5.17)^+^
Meditation	91	(9.4)	1.60	(0.95–2.70)	24	(8.8)	1.50	(0.80–2.82)	33	(11.4)	1.95	(1.07–3.54)^+^	18	(5.9)	1		19	(13.7)	2.32	(1.18–4.56)^+^
Alternative massage	49	(5.1)	2.76	(1.09–6.99)^+^	5	(1.8)	1		14	(4.8)	2.64	(0.94–7.44)	15	(4.9)	2.67	(0.96–7.43)	8	(5.7)	3.13	(1.01–9.75)^+^
Cranio sacral therapy	45	(4.6)	2.53	(0.99–6.44)	5	(1.8)	1		8	(2.8)	1.51	(0.49–4.68)	0	(0.0)	No data	No data	17	(12.2)	6.65	(2.40–18.41)^+^
Other supplements (probiotics, superfood, protein supplement, antioxidants, alkaline supplement, colloid silver, enzymes, fiber supplement, amino acid supplement, glucosamine, and mitochondrial energy optimizer, Gelée Royale LDN)	42	(4.3)	2.01	(0.62–6.58)	10	(3.7)	1.70	(0.46–6.29)	28	(9.7)	4.50	(1.35–15.07)^+^	15	(4.9)	2.27	(0.65–7.97)	3	(2.2)	1	
Healing	41	(4.2)	6.49	(1.56–26.98)^+^	20	(7.3)	11.25	(2.61–48.57)^+^	17	(5.9)	9.03	(2.07–39.44)^+^	2	(0.6)	1		17	(12.2)	18.71	(4.26–82.11)^+^
Homeopathy	28	(2.9)	1.67	(0.64–4.36)	15	(5.5)	3.18	(1.14–8.86)^+^	5	(1.7)	1		10	(3.3)	1.88	(0.64–5.57)	7	(5.0)	2.90	(0.90–9.30)
Amalgam removal	22	(2.3)	1		11	(4.0)	1.78	(0.85–3.71)	8	(2.8)	1.22	(0.54–2.77)	8	(2.6)	1.15	(0.51–2.61)	7	(5.0)	2.21	(0.93–5.28)
Qi gong	18	(1.8)	1		9	(3.3)	1.78	(0.79–4.00)	30	(10.4)	5.60	(3.07–10.19)^+^	0	(0.0)	No data	No data	0	(0.0)	No data	No data
Naprapathy	0	(0.0)	No data	No data	14	(5.1)	3.94	(1.28–12.11)^+^	11	(3.8)	2.92	(0.92–9.28)	4	(1.3)	1		0	(0.0)	No data	No data
CAM treatments with prevalence of use below 5% in all countries *Denmark*: Tai Chi, kinesiology, Feldenkrais method, osteopathy, hypnosis, orthomolecular treatment, cannabis, vacuum therapy, body SDS therapy, thought field therapy, Ayurvedic medicine, Birgitta Brunes' treatment, CCSVI surgery*, and alternative psychotherapy *Norway*: Tai Chi, Feldenkrais method, kinesiology, osteopathy, orthomolecular medicine, bioresonance/quantum medical treatment, Mensendick treatment, aroma therapy, thought field therapy, Ayurvedic medicine, Birgitta Brunes' treatment, CCSVI surgery, and alternative psycho therapy *Sweden*: Tai Chi, Feldenkrais method, osteopathy, kinesiology, hypnosis, bioresonance/quantum medical treatment, antroposofic medicine, Birgitta Brunes' treatment, CCSVI surgery, and alternative psychotherapy *Finland*: Tai Chi, colonic therapy, kinesiology, cannabis, lymph therapy, osteopathy, CCSVI surgery, and alternative psychotherapy	34	(3.5)	1.19	(0.57–2.52)	16	(5.9)	2.0	(0.87–4.60)	14	(4.8)	1.65	(0.70–3.87)	9	(2.9)	1		5	(3.6)	1.22	(0.40–3.71)
*Iceland*: Tai Chi, aroma therapy, Feldenkrais method, Birgitta Brunes' treatment, CCSVI surgery, and alternative psycho therapy																				
Use of one or more use of one or more energetic CAM modalities (including traditional Chinese acupuncture, reflexology, shiatsu massage, healing massage, cranial sacral therapy, healing, homeopathy, spritual psycho therapy, Qi gong, Tai Chi, and kinesiology)	223	(23.1)	2.14	(1.45–3.15)^+^	56	(20.6)	1.91	(1.21–3.02)^+^	48	(16.7)	(1.55)	(0.98–2.47)	33	(10.8)	1		43	(30.9)	2.87	(1.75–4.71)
Number of CAM modalities in use	29				29				26				22				20			

*It can be discussed whether CCSVI surgery should be classified as a CAM modality. Though it is not a “classic” CAM modality, we have chosen to include it in this table, based on the broad definition of CAM used in this study as an intervention not generally considered part of conventional medicine.

“^+^”signifies that the prevalence is significantly higher than the lowest prevalence. Significance has been determined by interpretation of the 95% confidence intervals (CIs) (if 1 is contained in the CI, *P* < 0.05).

**Table 3:** Overview of conventional modalities combined with CAM treatment among people with MS in the Nordic countries.

Conventional modality	Denmark (*n* = 967)				Norway (*n* = 272)				Sweden (*n* = 288)				Finland (*n* = 306)				Iceland (*n* = 139)			
	*n*	(%)	OR	(CI)	*n*	(%)	OR	(CI)	*n*	(%)	OR	(CI)	*n*	(%)	OR	(CI)	*n*	(%)	OR	(CI)
Physical therapy	582	(60.2)	1.66	(1.29–2.12)^+^	157	57.7	1.59	(1.18–2.15)^+^	104	(36.1)	1		148	(48.3)	1.33	(0.99–1.80)	75	(53.9)	1.49	(1.04–2.14)^+^
Prescription medicine	567	(58.6)	1.35	(1.06–1,71)^+^	118	43.4	1		173	(60.1)	1.38	(1.03–1.84)^+^	226	(73.8)	1.70	(1.29–2.24)^+^	81	(58.3)	1.30	(0.94–1.90)
Non prescription medicine	492	(50.9)	2.07	(1.40–3.06)^+^	87	31.9	1.30	(0.83–2.04)	120	(41.7)	1.70	(1.11–2.62)^+^	121	(39.5)	1.61	(1.05–2.48)^+^	34	(24.5)	1	
Conventional massage	145	(15.0)	1.35	(0.89–1.05)	30	11.0	1		73	(25.3)	2.29	(1.45–3.62)^+^	79	(25.8)	2.34	(1.49–3.67)^+^	27	(19.4)	1.76	(1.00–3.07)^+^
Therapeutic horseback riding	138	(14.3)	6.59	(2.07–20.99)^+^	7	2.6	1.19	(0.30–4.68)	12	(4.2)	1.93	(0.53–6.95)	14	(4.6)	2.11	(0.59–7.49)	3	(2.2)	1	
Psychology/ conventional psycho therapy	105	(10.9)	1.63	(0.97–2.74)	18	6.6	1		26	(9.0)	1.36	(0.73–2.54)	23	(7.5)	1.13	(0.60–2.14)	12	(8.6)	1.30	(0.61–2.78)
Chiropractics	73	(7.5)	3.84	(1.65–8.91)^+^	33	12.1	6.18	(2.55–14.99)^+^	13	(4.5)	2.30	(0.86–6.13)	6	(2.0)	1		11	(7.9)	4.06	(1.46–11.13)^+^
Occupational therapy	24	(2.5)	7.13	(0.96–52.95)	14	5.1	14.82	(1.93–113.94)^+^	1	(0.3)	1		14	(4.6)	13.17	(1.72–100.84)^+^	12	(8.6)	24.86	(3.20–193.14)^+^
Others (hot water exercise, electrical stimulation, and laser therapy)	14	(1.4)	1		7	2.6	1.78	(0.71–4.45)	8	(2.8)	1.92	(0.79–4.62)	7	(2.3)	1.58	(0.63–3.95)	6	(4.3)	2.98	(1.12–7.90)^+^
No conventional treatment	102	(10.5)	1.11	(0.72–1.71)	50	18.4	1.93	(1.19–3.15)^+^	51	(17.7)	1.86	(1.15–3.03)^+^	29	(9.5)	1		20	(14.4)	1.51	(0.83–2.77)

“^+^”signifies that the prevalence is significantly higher than the lowest prevalence. Significance has been determined by interpretation of the 95% confidence intervals (CIs) (if 1 is contained in the CI, *P* < 0.05).

**Table 4:** Overview of symptoms/health issues addressed by the use of CAM among people with MS in the Nordic countries.

Symptom/health issue addressed by use of CAM	Denmark (*n* = 967)				Norway (*n* = 272)				Sweden (*n* = 288)				Finland (*n* = 306)				Iceland (*n* = 139)			
	*n*	(%)	OR	(CI)	*n*	(%)	OR	(CI)	*n*	(%)	OR	(CI)	*n*	(%)	OR	(CI)	*n*	(%)	OR	(CI)
To strengthen the body in general	629	(65.0)	1.44	(1.15–1.81)^+^	174	(63.9)	1.42	(1.08–1.87)^+^	161	(55.9)	1.24	(0.94–1.64)	138	(45.0)	1		77	(55.3)	1.23	(0.87–1.73)
To improve well-being	510	(52.7)	1.35	(1.05–1.73)^+^	106	(38.9)	1		151	(52.4)	1.35	(0.99–1.81)	207	(67.6)	1.73	(1.30–2.31)^+^	100	(71.9)	1.84	(1.31–2.60)^+^
Fatigue/lack of energy	401	(41.5)	1.02	(0.80–1.31)	139	(51.1)	1.26	(0.94–1.71)	116	(40.2)	1		135	(44.1)	1.09	(0.81–1.47)	91	(65.4)	1.62	(1.15–2.28)^+^
To prevent symptoms	351	(36.3)	1.34	(1.01–1.77)^+^	92	(33.8)	1.25	(0.89–1.76)	78	(27.0)	1		104	(33.9)	1.25	(0.89–1.75)	54	(38.8)	1.43	(0.96–2.14)
Body pain	210	(21.7)	1.14	(0.83–1.57)	84	(30.8)	1.62	(1.12–2.36)^+^	72	(25.0)	1.31	(0.90–1.93)	58	(18.9)	1		64	(46.0)	2.43	(1.61–3.65)^+^
To improve the body's muscle strength	192	(19.9)	1.50	(1.04–2.18)^+^	50	(18.3)	1.39	(0.89–2.19)	38	(13.1)	1		43	(14.0)	1.06	(0.67–1.70)	55	(39.5)	2.99	(1.89–4.75)^+^
Problems with balance	189	(19.5)	1.53	(1.06–2.21)^+^	50	(18.3)	1.44	(0.92–2.26)	46	(15.9)	1.25	(0.79–1.98)	39	(12.7)	1		42	(30.2)	2.37	(1.46–3.83)^+^
To reduce the frequency of attacks	188	(19.4)	5.93	(3.10–11.36)^+^	33	(12.1)	3.71	(1.79–7.67)^+^	22	(7.6)	2.34	(1.09–5.02)^+^	10	(3.2)	1		39	(28.0)	8.58	(4.17–17.69)^+^
Spasms/tensions/cramps	176	(18.2)	1.08	(0.77–1.53)	56	(20.5)	1.23	0.81–1.88)	48	(16.6)	1		69	(22.5)	1.35	(0.91–2.02)	26	(18.7)	1.12	(0.67–1.88)
Sensing disorders	138	(14.3)	2.56	(1.50–4.37)^+^	21	(7.7)	1.39	(0.71–2.72)	16	(5.5)	1		24	(7.8)	1.41	(0.74–2.71)	24	(17.2)	3.11	(1.59–6.04)^+^
Indigestion/problems with the intestinal system	136	(14.1)	1		48	(17.6)	1.25	(0.88–1.79)	64	(22.2)	1.58	(1.14–2.19)^+^	60	(19.6)	1.39	(1.00–1.93)^+^	36	(25.8)	1.84	(1.23–2.77)^+^
Problems with bladder/urination	119	(12.3)	1.29	(0.84–1.98)	49	(18.0)	1.90	(1.16–3.09)^+^	32	(11.1)	1.17	(0.69–1.98)	29	(9.4)	1		32	(23.0)	2.42	(1.41–4.17)^+^
Headache	115	(11.9)	1.45	(0.92–2.28)	36	(13.2)	1.62	(0.94–2.76)	24	(8.3)	1.02	(0.57–1.82)	25	(8.1)	1		34	(24.4)	2.99	(1.72–5.21)^+^
General discomfort	113	(11.7)	1.12	(0.73–1.71)	43	(15.8)	1.52	(0.93–2.49)	30	(10.4)	1		45	(14.7)	1.41	(0.87–2.30)	44	(31.6)	3.04	(1.83–5.04)^+^
Problems with walking	105	(10.9)	1.08	(0.59–1.93)	28	(10.2)	1.02	(0.52–2.00)	31	(10.7)	1.09	(0.55–2.07)	38	(12.4)	1.23	(0.65–2.35)	14	(10.0)	1	
Cognitive problems	83	(8.6)	2.38	(1.25–4.53)^+^	24	(8.8)	2.45	(1.18–5.10)^+^	21	(7.2)	2.03	(0.96–4.28)	11	(3.5)	1		28	(20.1)	5.60	(2.71–11.58)^+^
Psychological problems	82	(8.5)	2.59	(1.33–5.06)^+^	26	(9.5)	2.92	(1.38–6.18)^+^	17	(5.9)	1.81	(0.81–4.01)	10	(3.2)	1		27	(19.4)	5.94	(2.80–12.62)^+^
Dizziness	82	(8.5)	3.69	(1.69–8.09)^+^	20	(7.3)	3.21	(1.34–7.72)^+^	11	(3.8)	1.67	(0.64–4.37)	7	(2.2)	1		23	(16.5)	7.23	(3.03–17.26)^+^
Problems with coordination/shaking	67	(6.9)	1.88	(0.95–3.70)	10	(3.6)	1		16	(5.5)	1.51	(0.67–3.39)	16	(5.2)	1.33	(0.59–3.02)	21	(15.1)	4.11	(1.88–8.96)^+^
Visual disorders	49	(5.1)	1.32	(0.68–2.58)	11	(4.0)	1.06	(0.45–2.48)	11	(3.8)	1		13	(4.2)	1.11	(0.49–2.52)	22	(15.8)	4.14	(1.95–8.78)^+^
Paralysis	26	(2.7)	1.64	(0.62–4.31)	7	(2.5)	1.57	(0.49–5.02)	7	(2.4)	1.48	(0.46–4.7)	5	(1.6)	1		10	(7.1)	4.40	(1.47–13.12)^+^
Problems with speech, chewing, and swallowing	22	(2.3)	1		8	(2.9)	1.29	(0.57–2.94)	10	(3.4)	1.53	(0.72–3.27)	8	(2.6)	1.15	(0.51–2.61)	16	(11.5)	5.07	(2.59–9.89)^+^
Symptoms/health issues addressed by less than 2% in all countries (cold hands and feet, nails, hair, PMS, itching, menopause, weight, sleep, eczema, allergies, lack of appetite, canker sores, blood sugar instability, pregnancy, detox, oedema, stress, and sexual problems)	50	(5.2)	1.17	(0.61–2.23)	12	(4.4)	1		22	(7.63)	1.73	(0.84–3.56)	19	(6.20)	1.41	(0.67–2.95)	9	(6.4)	1.46	(0.60–3.56)
Addressing of one or more nonspecific/preventive symptoms/health issues (including strengthening the body in general, improving well-being, preventing symptoms, improving the body's muscle strength, and reducing the frequency of attacks and general discomfort)	797	82.2	1.04	(0.85–1.26)	222	81.6	1.03	(0.80–1.32)	228	79.2	1		256	83.7	1.06	(0.83–1.34)	118	84.9	1.07	(0.79–1.45)

“^+^”signifies that the prevalence is significantly higher than the lowest prevalence. Significance has been determined by interpretation of the 95% confidence intervals (CIs) (if 1 is contained in the CI, *P* < 0.05).
